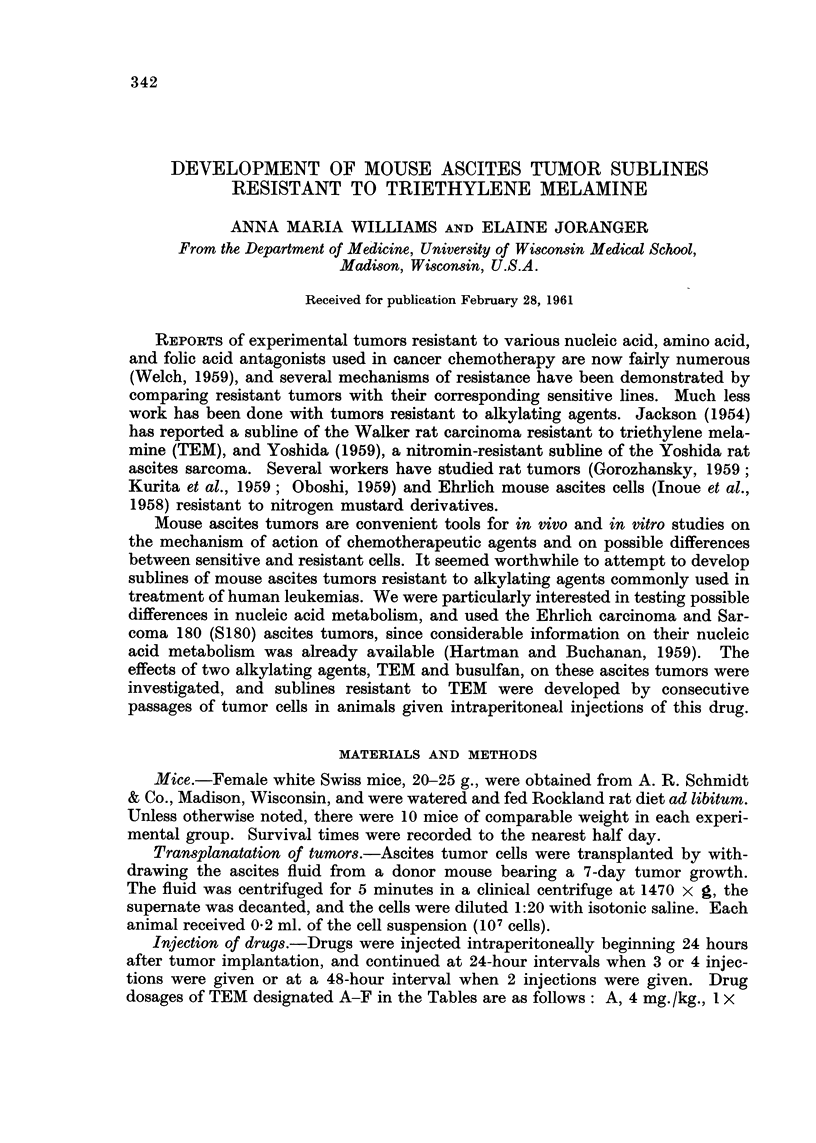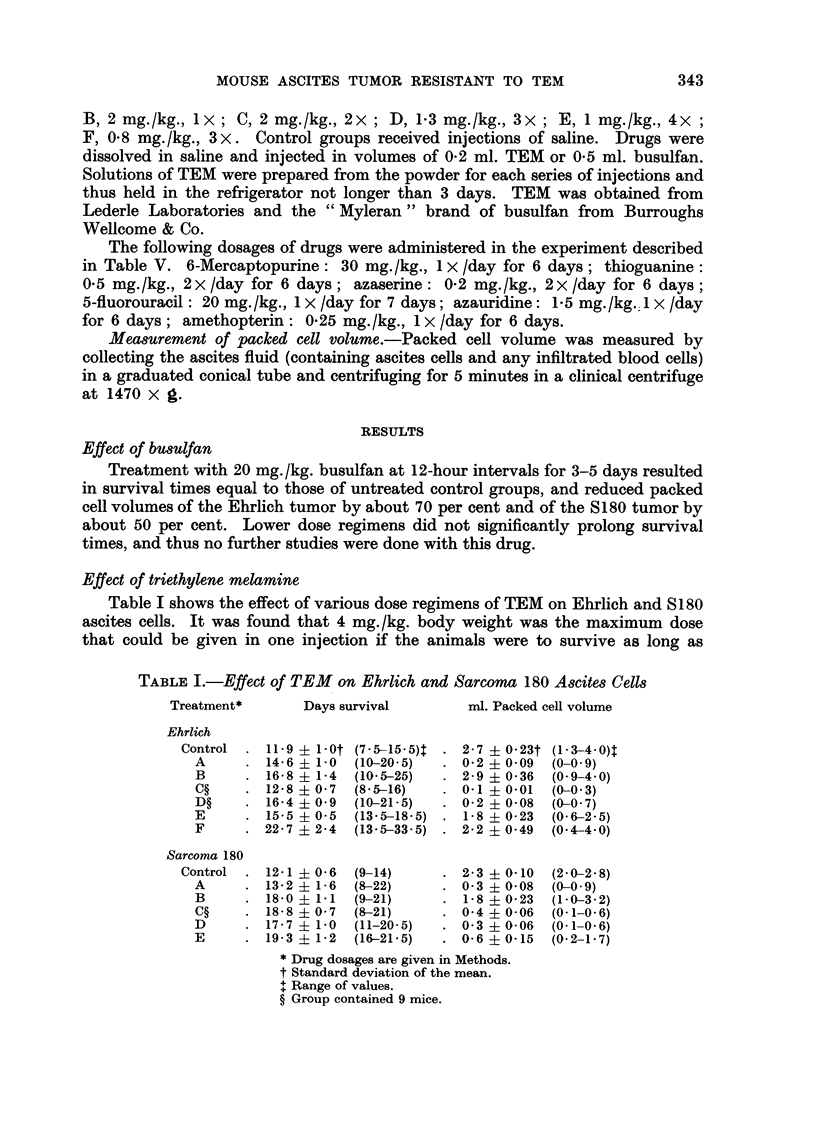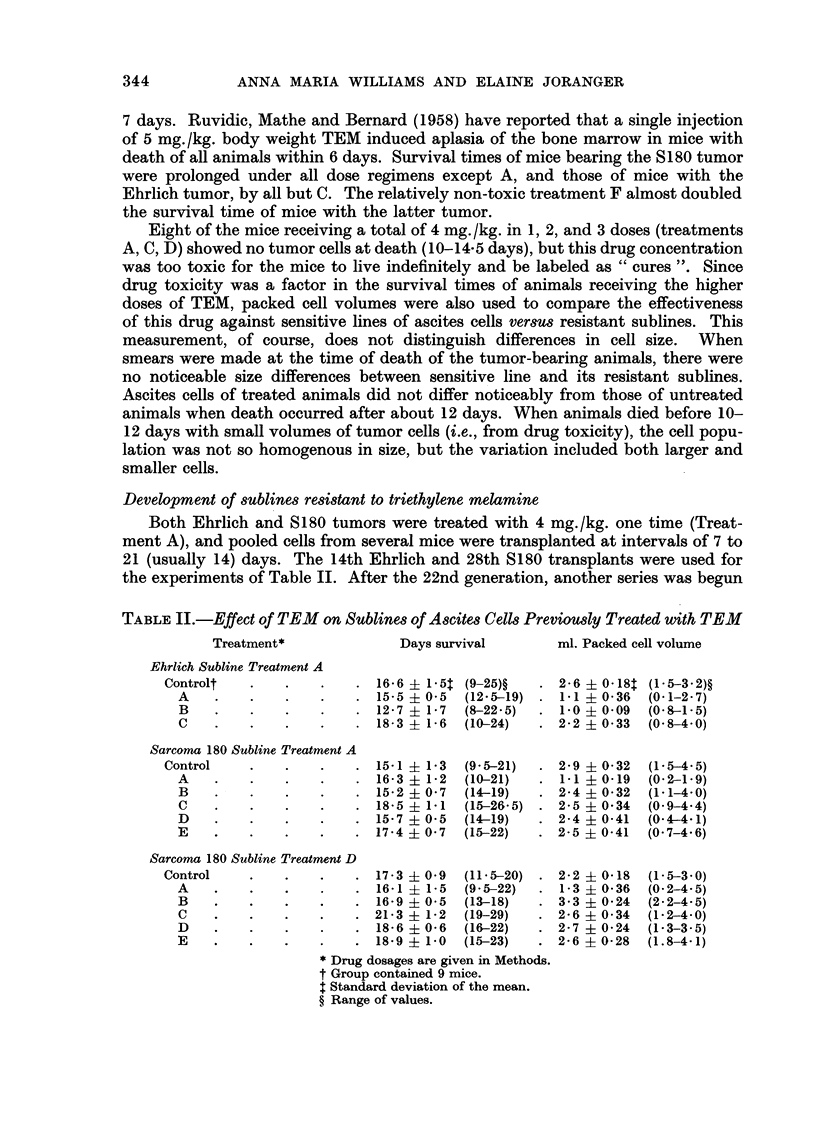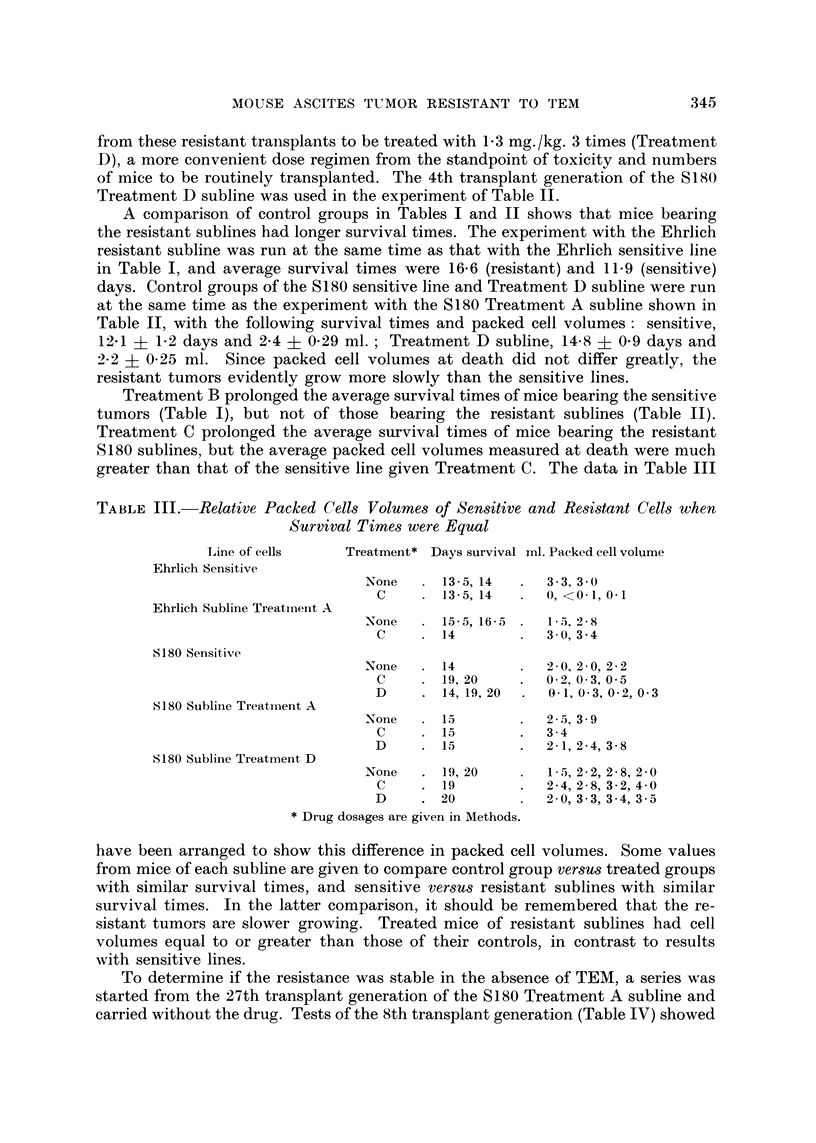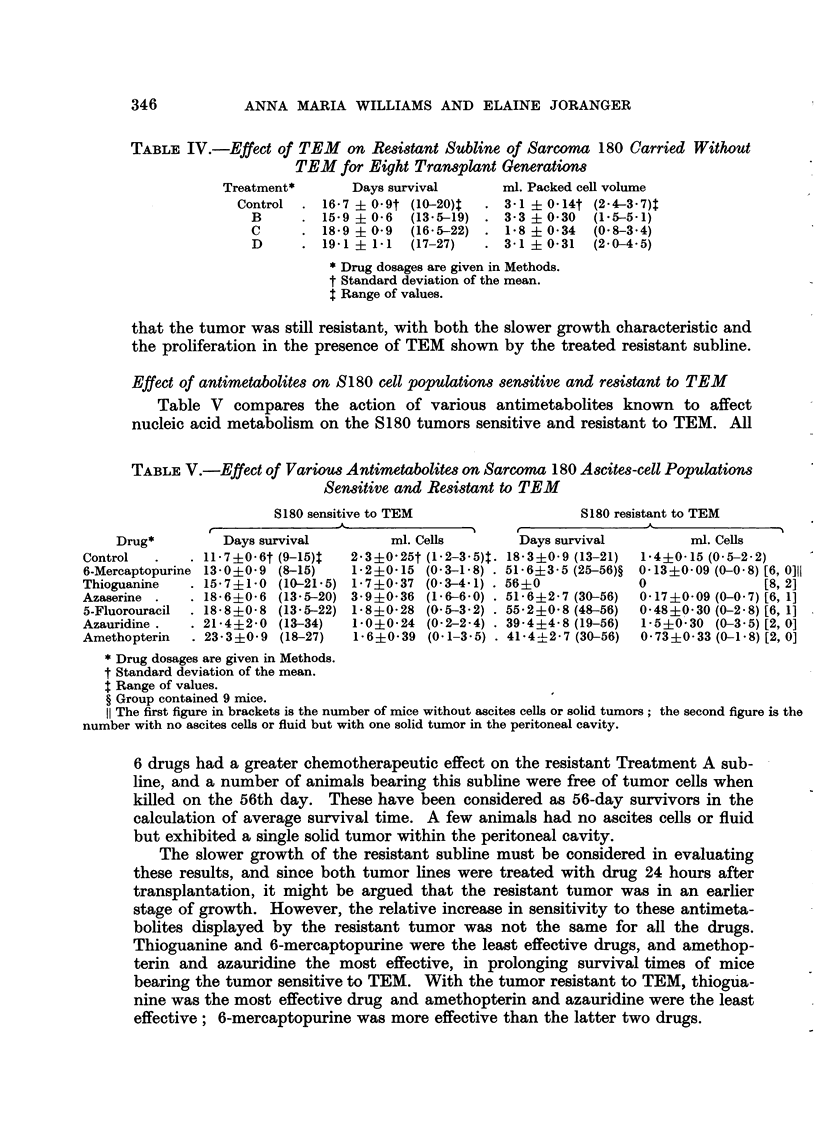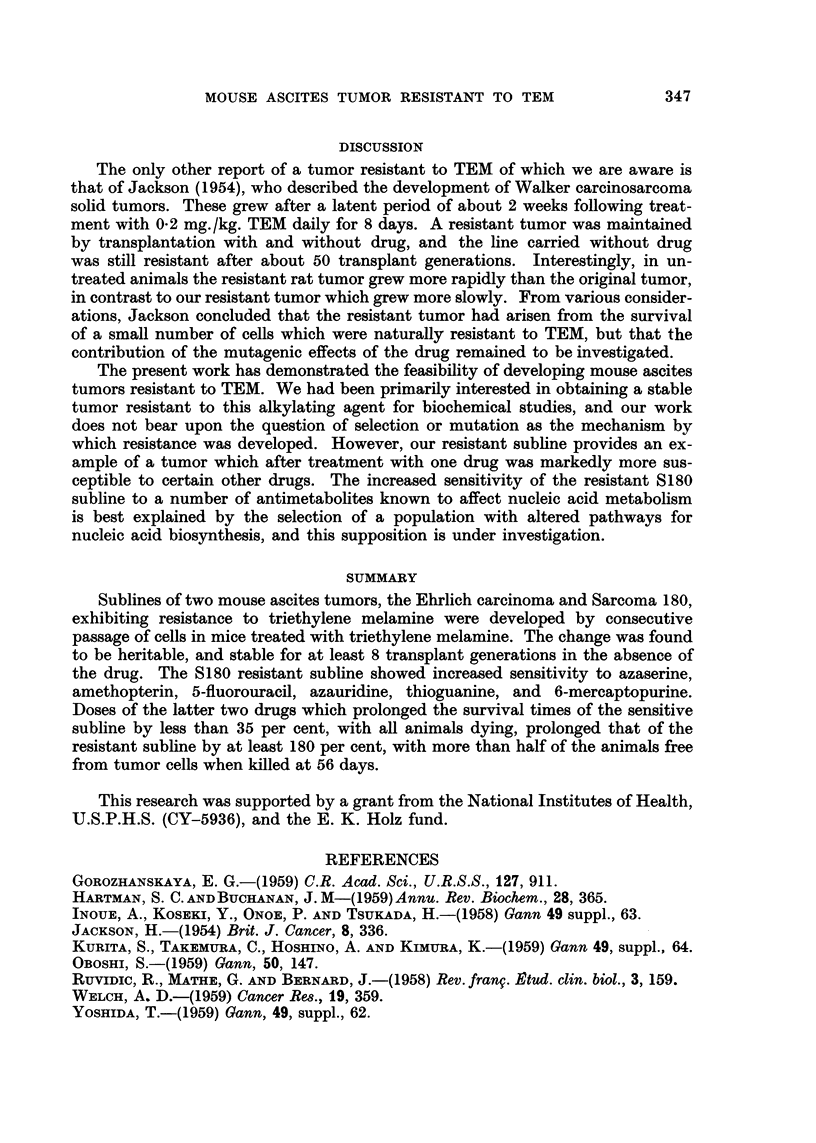# Development of Mouse Ascites Tumour Sublines Resistant to Triethylene Melamine

**DOI:** 10.1038/bjc.1961.43

**Published:** 1961-06

**Authors:** Anna Maria Williams, Elaine Joranger


### DEVELOPMENT OF MOUSE ASCITES TUMOR SUBLINES RESISTANT TO TRIETHYLENE MELAMINE

ANNA MARIA WILLIAMS AND ELAINE JORANGER
*From the Department of Medicine, University of Wisconsin Medical School, Madison, Wisconsin, U.S.A.*

Received for publication February 28, 1961

REPORTS of experimental tumors resistant to various nucleic acid, amino acid, and folic acid antagonists used in cancer chemotherapy are now fairly numerous (Welch, 1959), and several mechanisms of resistance have been demonstrated by comparing resistant tumors with their corresponding sensitive lines. Much less work has been done with tumors resistant to alkylating agents. Jackson (1954) has reported a subline of the Walker rat carcinoma resistant to triethylene melamine (TEM), and Yoshida (1959), a nitromin-resistant subline of the Yoshida rat ascites sarcoma. Several workers have studied rat tumors (Gorozhansky, 1959 ; Kurita *et al.*, 1959 ; Oboshi, 1959) and Ehrlich mouse ascites cells (Inoue *et al.*, 1958) resistant to nitrogen mustard derivatives.

Mouse ascites tumors are convenient tools for *in vivo* and *in vitro* studies on the mechanism of action of chemotherapeutic agents and on possible differences between sensitive and resistant cells. It seemed worthwhile to attempt to develop sublines of mouse ascites tumors resistant to alkylating agents commonly used in treatment of human leukemias. We were particularly interested in testing possible differences in nucleic acid metabolism, and used the Ehrlich carcinoma and Sarcoma 180 (S180) ascites tumors, since considerable information on their nucleic acid metabolism was already available (Hartman and Buchanan, 1959). The effects of two alkylating agents, TEM and busulfan, on these ascites tumors were investigated, and sublines resistant to TEM were developed by consecutive passages of tumor cells in animals given intraperitoneal injections of this drug.

### MATERIALS AND METHODS

*Mice.*—Female white Swiss mice, 20–25 g., were obtained from A. R. Schmidt & Co., Madison, Wisconsin, and were watered and fed Rockland rat diet *ad libitum.* Unless otherwise noted, there were 10 mice of comparable weight in each experimental group. Survival times were recorded to the nearest half day.

*Transplanatation of tumors.*—Ascites tumor cells were transplanted by withdrawing the ascites fluid from a donor mouse bearing a 7-day tumor growth. The fluid was centrifuged for 5 minutes in a clinical centrifuge at 1470 × g, the supernate was decanted, and the cells were diluted 1:20 with isotonic saline. Each animal received 0·2 ml. of the cell suspension ($10^7$ cells).

*Injection of drugs.*—Drugs were injected intraperitoneally beginning 24 hours after tumor implantation, and continued at 24-hour intervals when 3 or 4 injections were given or at a 48-hour interval when 2 injections were given. Drug dosages of TEM designated A–F in the Tables are as follows : A, 4 mg./kg., 1×

B, 2 mg./kg., 1× ; C, 2 mg./kg., 2× ; D, 1·3 mg./kg., 3× ; E, 1 mg./kg., 4× ; F, 0·8 mg./kg., 3×. Control groups received injections of saline. Drugs were dissolved in saline and injected in volumes of 0·2 ml. TEM or 0·5 ml. busulfan. Solutions of TEM were prepared from the powder for each series of injections and thus held in the refrigerator not longer than 3 days. TEM was obtained from Lederle Laboratories and the "Myleran" brand of busulfan from Burroughs Wellcome & Co.

The following dosages of drugs were administered in the experiment described in Table V. 6-Mercaptopurine: 30 mg./kg., 1× /day for 6 days; thioguanine: 0·5 mg./kg., 2× /day for 6 days; azaserine: 0·2 mg./kg., 2× /day for 6 days; 5-fluorouracil: 20 mg./kg., 1× /day for 7 days; azauridine: 1·5 mg./kg., 1× /day for 6 days; amethopterin: 0·25 mg./kg., 1× /day for 6 days.

*Measurement of packed cell volume.*—Packed cell volume was measured by collecting the ascites fluid (containing ascites cells and any infiltrated blood cells) in a graduated conical tube and centrifuging for 5 minutes in a clinical centrifuge at 1470 × g.

### RESULTS

### *Effect of busulfan*

Treatment with 20 mg./kg. busulfan at 12-hour intervals for 3–5 days resulted in survival times equal to those of untreated control groups, and reduced packed cell volumes of the Ehrlich tumor by about 70 per cent and of the S180 tumor by about 50 per cent. Lower dose regimens did not significantly prolong survival times, and thus no further studies were done with this drug.

### *Effect of triethylene melamine*

Table I shows the effect of various dose regimens of TEM on Ehrlich and S180 ascites cells. It was found that 4 mg./kg. body weight was the maximum dose that could be given in one injection if the animals were to survive as long as

TABLE I.—*Effect of TEM on Ehrlich and Sarcoma* 180 *Ascites Cells*

| Treatment* | Days survival | | ml. Packed cell volume | |
|---|---|---|---|---|
| *Ehrlich* | | | | |
| Control | 11·9 ± 1·0† | (7·5–15·5)‡ | 2·7 ± 0·23† | (1·3–4·0)‡ |
| A | 14·6 ± 1·0 | (10–20·5) | 0·2 ± 0·09 | (0–0·9) |
| B | 16·8 ± 1·4 | (10·5–25) | 2·9 ± 0·36 | (0·9–4·0) |
| C§ | 12·8 ± 0·7 | (8·5–16) | 0·1 ± 0·01 | (0–0·3) |
| D§ | 16·4 ± 0·9 | (10–21·5) | 0·2 ± 0·08 | (0–0·7) |
| E | 15·5 ± 0·5 | (13·5–18·5) | 1·8 ± 0·23 | (0·6–2·5) |
| F | 22·7 ± 2·4 | (13·5–33·5) | 2·2 ± 0·49 | (0·4–4·0) |
| *Sarcoma* 180 | | | | |
| Control | 12·1 ± 0·6 | (9–14) | 2·3 ± 0·10 | (2·0–2·8) |
| A | 13·2 ± 1·6 | (8–22) | 0·3 ± 0·08 | (0–0·9) |
| B | 18·0 ± 1·1 | (9–21) | 1·8 ± 0·23 | (1·0–3·2) |
| C§ | 18·8 ± 0·7 | (8–21) | 0·4 ± 0·06 | (0·1–0·6) |
| D | 17·7 ± 1·0 | (11–20·5) | 0·3 ± 0·06 | (0·1–0·6) |
| E | 19·3 ± 1·2 | (16–21·5) | 0·6 ± 0·15 | (0·2–1·7) |

* Drug dosages are given in Methods.
† Standard deviation of the mean.
‡ Range of values.
§ Group contained 9 mice.

7 days. Ruvidic, Mathe and Bernard (1958) have reported that a single injection of 5 mg./kg. body weight TEM induced aplasia of the bone marrow in mice with death of all animals within 6 days. Survival times of mice bearing the S180 tumor were prolonged under all dose regimens except A, and those of mice with the Ehrlich tumor, by all but C. The relatively non-toxic treatment F almost doubled the survival time of mice with the latter tumor.

Eight of the mice receiving a total of 4 mg./kg. in 1, 2, and 3 doses (treatments A, C, D) showed no tumor cells at death (10–14·5 days), but this drug concentration was too toxic for the mice to live indefinitely and be labeled as " cures ". Since drug toxicity was a factor in the survival times of animals receiving the higher doses of TEM, packed cell volumes were also used to compare the effectiveness of this drug against sensitive lines of ascites cells *versus* resistant sublines. This measurement, of course, does not distinguish differences in cell size. When smears were made at the time of death of the tumor-bearing animals, there were no noticeable size differences between sensitive line and its resistant sublines. Ascites cells of treated animals did not differ noticeably from those of untreated animals when death occurred after about 12 days. When animals died before 10–12 days with small volumes of tumor cells (*i.e.*, from drug toxicity), the cell population was not so homogenous in size, but the variation included both larger and smaller cells.

*Development of sublines resistant to triethylene melamine*

Both Ehrlich and S180 tumors were treated with 4 mg./kg. one time (Treatment A), and pooled cells from several mice were transplanted at intervals of 7 to 21 (usually 14) days. The 14th Ehrlich and 28th S180 transplants were used for the experiments of Table II. After the 22nd generation, another series was begun

TABLE II.—*Effect of TEM on Sublines of Ascites Cells Previously Treated with TEM*

| Treatment* | Days survival | | ml. Packed cell volume | |
|---|---|---|---|---|
| *Ehrlich Subline Treatment A* | | | | |
| Control† | 16·6 ± 1·5‡ | (9–25)§ | 2·6 ± 0·18‡ | (1·5–3·2)§ |
| A | 15·5 ± 0·5 | (12·5–19) | 1·1 ± 0·36 | (0·1–2·7) |
| B | 12·7 ± 1·7 | (8–22·5) | 1·0 ± 0·09 | (0·8–1·5) |
| C | 18·3 ± 1·6 | (10–24) | 2·2 ± 0·33 | (0·8–4·0) |
| *Sarcoma* 180 *Subline Treatment A* | | | | |
| Control | 15·1 ± 1·3 | (9·5–21) | 2·9 ± 0·32 | (1·5–4·5) |
| A | 16·3 ± 1·2 | (10–21) | 1·1 ± 0·19 | (0·2–1·9) |
| B | 15·2 ± 0·7 | (14–19) | 2·4 ± 0·32 | (1·1–4·0) |
| C | 18·5 ± 1·1 | (15–26·5) | 2·5 ± 0·34 | (0·9–4·4) |
| D | 15·7 ± 0·5 | (14–19) | 2·4 ± 0·41 | (0·4–4·1) |
| E | 17·4 ± 0·7 | (15–22) | 2·5 ± 0·41 | (0·7–4·6) |
| *Sarcoma* 180 *Subline Treatment D* | | | | |
| Control | 17·3 ± 0·9 | (11·5–20) | 2·2 ± 0·18 | (1·5–3·0) |
| A | 16·1 ± 1·5 | (9·5–22) | 1·3 ± 0·36 | (0·2–4·5) |
| B | 16·9 ± 0·5 | (13–18) | 3·3 ± 0·24 | (2·2–4·5) |
| C | 21·3 ± 1·2 | (19–29) | 2·6 ± 0·34 | (1·2–4·0) |
| D | 18·6 ± 0·6 | (16–22) | 2·7 ± 0·24 | (1·3–3·5) |
| E | 18·9 ± 1·0 | (15–23) | 2·6 ± 0·28 | (1.8–4·1) |

* Drug dosages are given in Methods.
† Group contained 9 mice.
‡ Standard deviation of the mean.
§ Range of values.

from these resistant transplants to be treated with 1·3 mg./kg. 3 times (Treatment D), a more convenient dose regimen from the standpoint of toxicity and numbers of mice to be routinely transplanted. The 4th transplant generation of the S180 Treatment D subline was used in the experiment of Table II.

A comparison of control groups in Tables I and II shows that mice bearing the resistant sublines had longer survival times. The experiment with the Ehrlich resistant subline was run at the same time as that with the Ehrlich sensitive line in Table I, and average survival times were 16·6 (resistant) and 11·9 (sensitive) days. Control groups of the S180 sensitive line and Treatment D subline were run at the same time as the experiment with the S180 Treatment A subline shown in Table II, with the following survival times and packed cell volumes: sensitive, 12·1 $\pm$ 1·2 days and 2·4 $\pm$ 0·29 ml.; Treatment D subline, 14·8 $\pm$ 0·9 days and 2·2 $\pm$ 0·25 ml. Since packed cell volumes at death did not differ greatly, the resistant tumors evidently grow more slowly than the sensitive lines.

Treatment B prolonged the average survival times of mice bearing the sensitive tumors (Table I), but not of those bearing the resistant sublines (Table II). Treatment C prolonged the average survival times of mice bearing the resistant S180 sublines, but the average packed cell volumes measured at death were much greater than that of the sensitive line given Treatment C. The data in Table III

TABLE III.—*Relative Packed Cells Volumes of Sensitive and Resistant Cells when Survival Times were Equal*

| Line of cells | Treatment* | Days survival | ml. Packed cell volume |
|---|---|---|---|
| Ehrlich Sensitive | | | |
| | None | 13·5, 14 | 3·3, 3·0 |
| | C | 13·5, 14 | 0, <0·1, 0·1 |
| Ehrlich Subline Treatment A | | | |
| | None | 15·5, 16·5 | 1·5, 2·8 |
| | C | 14 | 3·0, 3·4 |
| S180 Sensitive | | | |
| | None | 14 | 2·0, 2·0, 2·2 |
| | C | 19, 20 | 0·2, 0·3, 0·5 |
| | D | 14, 19, 20 | 0·1, 0·3, 0·2, 0·3 |
| S180 Subline Treatment A | | | |
| | None | 15 | 2·5, 3·9 |
| | C | 15 | 3·4 |
| | D | 15 | 2·1, 2·4, 3·8 |
| S180 Subline Treatment D | | | |
| | None | 19, 20 | 1·5, 2·2, 2·8, 2·0 |
| | C | 19 | 2·4, 2·8, 3·2, 4·0 |
| | D | 20 | 2·0, 3·3, 3·4, 3·5 |

* Drug dosages are given in Methods.

have been arranged to show this difference in packed cell volumes. Some values from mice of each subline are given to compare control group *versus* treated groups with similar survival times, and sensitive *versus* resistant sublines with similar survival times. In the latter comparison, it should be remembered that the resistant tumors are slower growing. Treated mice of resistant sublines had cell volumes equal to or greater than those of their controls, in contrast to results with sensitive lines.

To determine if the resistance was stable in the absence of TEM, a series was started from the 27th transplant generation of the S180 Treatment A subline and carried without the drug. Tests of the 8th transplant generation (Table IV) showed

TABLE IV.—*Effect of TEM on Resistant Subline of Sarcoma 180 Carried Without TEM for Eight Transplant Generations*

| Treatment* | Days survival | | ml. Packed cell volume | |
|---|---|---|---|---|
| Control | 16·7 ± 0·9† | (10–20)‡ | 3·1 ± 0·14† | (2·4–3·7)‡ |
| B | 15·9 ± 0·6 | (13·5–19) | 3·3 ± 0·30 | (1·5–5·1) |
| C | 18·9 ± 0·9 | (16·5–22) | 1·8 ± 0·34 | (0·8–3·4) |
| D | 19·1 ± 1·1 | (17–27) | 3·1 ± 0·31 | (2·0–4·5) |

* Drug dosages are given in Methods.
† Standard deviation of the mean.
‡ Range of values.

that the tumor was still resistant, with both the slower growth characteristic and the proliferation in the presence of TEM shown by the treated resistant subline.

*Effect of antimetabolites on S180 cell populations sensitive and resistant to TEM*

Table V compares the action of various antimetabolites known to affect nucleic acid metabolism on the S180 tumors sensitive and resistant to TEM. All

TABLE V.—*Effect of Various Antimetabolites on Sarcoma 180 Ascites-cell Populations Sensitive and Resistant to TEM*

| | S180 sensitive to TEM | | | | S180 resistant to TEM | | | | |
|---|---|---|---|---|---|---|---|---|---|
| Drug* | Days survival | | ml. Cells | | Days survival | | ml. Cells | | |
| Control | 11·7±0·6† | (9–15)‡ | 2·3±0·25† | (1·2–3·5)‡ | 18·3±0·9 | (13–21) | 1·4±0·15 | (0·5–2·2) | |
| 6-Mercaptopurine | 13·0±0·9 | (8–15) | 1·2±0·15 | (0·3–1·8) | 51·6±3·5 | (25–56)§ | 0·13±0·09 | (0–0·8) | [6, 0]‖ |
| Thioguanine | 15·7±1·0 | (10–21·5) | 1·7±0·37 | (0·3–4·1) | 56±0 | | 0 | | [8, 2] |
| Azaserine | 18·6±0·6 | (13·5–20) | 3·9±0·36 | (1·6–6·0) | 51·6±2·7 | (30–56) | 0·17±0·09 | (0–0·7) | [6, 1] |
| 5-Fluorouracil | 18·8±0·8 | (13·5–22) | 1·8±0·28 | (0·5–3·2) | 55·2±0·8 | (48–56) | 0·48±0·30 | (0–2·8) | [6, 1] |
| Azauridine | 21·4±2·0 | (13–34) | 1·0±0·24 | (0·2–2·4) | 39·4±4·8 | (19–56) | 1·5±0·30 | (0–3·5) | [2, 0] |
| Amethopterin | 23·3±0·9 | (18–27) | 1·6±0·39 | (0·1–3·5) | 41·4±2·7 | (30–56) | 0·73±0·33 | (0–1·8) | [2, 0] |

* Drug dosages are given in Methods.
† Standard deviation of the mean.
‡ Range of values.
§ Group contained 9 mice.
‖ The first figure in brackets is the number of mice without ascites cells or solid tumors ; the second figure is the number with no ascites cells or fluid but with one solid tumor in the peritoneal cavity.

6 drugs had a greater chemotherapeutic effect on the resistant Treatment A subline, and a number of animals bearing this subline were free of tumor cells when killed on the 56th day. These have been considered as 56-day survivors in the calculation of average survival time. A few animals had no ascites cells or fluid but exhibited a single solid tumor within the peritoneal cavity.

The slower growth of the resistant subline must be considered in evaluating these results, and since both tumor lines were treated with drug 24 hours after transplantation, it might be argued that the resistant tumor was in an earlier stage of growth. However, the relative increase in sensitivity to these antimetabolites displayed by the resistant tumor was not the same for all the drugs. Thioguanine and 6-mercaptopurine were the least effective drugs, and amethopterin and azauridine the most effective, in prolonging survival times of mice bearing the tumor sensitive to TEM. With the tumor resistant to TEM, thioguanine was the most effective drug and amethopterin and azauridine were the least effective ; 6-mercaptopurine was more effective than the latter two drugs.

### DISCUSSION

The only other report of a tumor resistant to TEM of which we are aware is that of Jackson (1954), who described the development of Walker carcinosarcoma solid tumors. These grew after a latent period of about 2 weeks following treatment with 0·2 mg./kg. TEM daily for 8 days. A resistant tumor was maintained by transplantation with and without drug, and the line carried without drug was still resistant after about 50 transplant generations. Interestingly, in untreated animals the resistant rat tumor grew more rapidly than the original tumor, in contrast to our resistant tumor which grew more slowly. From various considerations, Jackson concluded that the resistant tumor had arisen from the survival of a small number of cells which were naturally resistant to TEM, but that the contribution of the mutagenic effects of the drug remained to be investigated.

The present work has demonstrated the feasibility of developing mouse ascites tumors resistant to TEM. We had been primarily interested in obtaining a stable tumor resistant to this alkylating agent for biochemical studies, and our work does not bear upon the question of selection or mutation as the mechanism by which resistance was developed. However, our resistant subline provides an example of a tumor which after treatment with one drug was markedly more susceptible to certain other drugs. The increased sensitivity of the resistant S180 subline to a number of antimetabolites known to affect nucleic acid metabolism is best explained by the selection of a population with altered pathways for nucleic acid biosynthesis, and this supposition is under investigation.

### SUMMARY

Sublines of two mouse ascites tumors, the Ehrlich carcinoma and Sarcoma 180, exhibiting resistance to triethylene melamine were developed by consecutive passage of cells in mice treated with triethylene melamine. The change was found to be heritable, and stable for at least 8 transplant generations in the absence of the drug. The S180 resistant subline showed increased sensitivity to azaserine, amethopterin, 5-fluorouracil, azauridine, thioguanine, and 6-mercaptopurine. Doses of the latter two drugs which prolonged the survival times of the sensitive subline by less than 35 per cent, with all animals dying, prolonged that of the resistant subline by at least 180 per cent, with more than half of the animals free from tumor cells when killed at 56 days.

This research was supported by a grant from the National Institutes of Health, U.S.P.H.S. (CY-5936), and the E. K. Holz fund.

### REFERENCES

Gorozhanskaya, E. G.—(1959) *C.R. Acad. Sci., U.R.S.S.*, **127**, 911.
Hartman, S. C. and Buchanan, J. M—(1959) *Annu. Rev. Biochem.*, **28**, 365.
Inoue, A., Koseki, Y., Onoe, P. and Tsukada, H.—(1958) *Gann* **49** suppl., 63.
Jackson, H.—(1954) *Brit. J. Cancer*, **8**, 336.
Kurita, S., Takemura, C., Hoshino, A. and Kimura, K.—(1959) *Gann* **49**, suppl., 64.
Oboshi, S.—(1959) *Gann*, **50**, 147.
Ruvidic, R., Mathe, G. and Bernard, J.—(1958) *Rev. franç. Étud. clin. biol.*, **3**, 159.
Welch, A. D.—(1959) *Cancer Res.*, **19**, 359.
Yoshida, T.—(1959) *Gann*, **49**, suppl., 62.